# Neurofibromatosis type 1-dependent alterations in mouse microglia function are not cell-intrinsic

**DOI:** 10.1186/s40478-023-01525-w

**Published:** 2023-03-09

**Authors:** Francesca Logiacco, Laura Cathleen Grzegorzek, Elizabeth C. Cordell, Oliver Popp, Philipp Mertins, David H. Gutmann, Helmut Kettenmann, Marcus Semtner

**Affiliations:** 1 Cellular Neurosciences, Max-Delbrück-Center for Molecular Medicine in the Helmholtz Association, 13125 Berlin, Germany; 2 Department of Neurology, Washington University School of Medicine, St. Louis, MO 63110 USA; 3 Institute of Cell Biology and Neurobiology, Charité–Universitätsmedizin Berlin, corporate member of Freie Universität Berlin, Humboldt-Universität Berlin, and Berlin Institute of Health, 10117 Berlin, Germany; 4 Proteomics Platform, Max-Delbrück-Center for Molecular Medicine in the Helmholtz Association, 13125 Berlin, Germany; 5 Shenzhen Institute of Advanced Technology, Chinese Academy of Sciences, Shenzhen, China

**Keywords:** Neurofibromin, NF1, Purinergic signaling, Sex differences, Microglia, Cortex, Genetically engineered mice

## Abstract

We previously discovered a sex-by-genotype defect in microglia function using a heterozygous germline knockout mouse model of Neurofibromatosis type 1 (*Nf1* ± mice), in which only microglia from male *Nf1* ± mice exhibited defects in purinergic signaling. Herein, we leveraged an unbiased proteomic approach to demonstrate that male, but not female, heterozygous *Nf1* ± microglia exhibit differences in protein expression, which largely reflect pathways involved in cytoskeletal organization. In keeping with these predicted defects in cytoskeletal function, only male *Nf1* ± microglia had reduced process arborization and surveillance capacity. To determine whether these microglial defects were cell autonomous or reflected adaptive responses to *Nf1* heterozygosity in other cells in the brain, we generated conditional microglia *Nf1*-mutant knockout mice by intercrossing *Nf1*^flox/flox^ with Cx3cr1-Cre^ER^ mice (*Nf1*^flox/wt^; Cx3cr1-Cre^ER^ mice, *Nf1*^MG^ ± mice). Surprisingly, neither male nor female *Nf1*^MG^ ± mouse microglia had impaired process arborization or surveillance capacity. In contrast, when *Nf1* heterozygosity was generated in neurons, astrocytes and oligodendrocytes by intercrossing *Nf1*^flox/flox^ with hGFAP-Cre mice (*Nf1*^flox/wt^; hGFAP-Cre mice, *Nf1*^GFAP^ ± mice), the microglia defects found in *Nf1* ± mice were recapitulated. Collectively, these data reveal that *Nf1* ± sexually dimorphic microglia abnormalities are likely not cell-intrinsic properties, but rather reflect a response to *Nf1* heterozygosity in other brain cells.

**Supplementary Information:**

The online version contains supplementary material available at 10.1186/s40478-023-01525-w.

## Introduction

Microglia are highly adaptive cells, comprising 5% of the cells in the central nervous system, which serve numerous functions critical to brain homeostasis and response to disease [43, 68]. Changes in microglia function can originate from cell-intrinsic alterations in their innate properties or reactions to environmental stimuli (cell-extrinsic). For example, APOE4 genotype confers transcriptomic and functional alterations in primary mouse microglia in vitro [41], while microglia-specific ApoE loss reduces A-beta plaque size in a mouse model of Alzheimer’s disease [28]. Conversely, neurons and macroglia (oligodendrocytes and astrocytes) can communicate with microglia to influence their migration and phagocytosis through the elaboration of paracrine factors (e.g., chemokines; [25, 52] and/or expression of cell surface proteins (e.g., CD47; [38]. This adaptability is underscored by multiple reports demonstrating that microglia can undergo changes in gene expression and function when they are analyzed in vitro relative to their in vivo state [6, 43, 49, 62]. In this fashion, understanding microglia function requires a consideration of both cell-autonomous and non-cell-autonomous properties.

We have previously demonstrated that microglia from mice heterozygous for a germline inactivating mutation in the Neurofibromatosis type 1 (NF1) gene exhibit sexually dimorphic impairments in purinergic function [17, 18]. Using in situ analysis of microglia function, we showed that microglia from male, but not female, *Nf1* ± mice exhibit impaired phagocytosis, ATP-evoked membrane currents, and lesion-induced process accumulation relative to their wild-type counterparts. While these results could be interpreted as revealing cell-intrinsic effects of *Nf1* mutation on microglia function, it is equally possible that the observed abnormalities result from indirect effects on microglia, operating through the impact of *Nf1* mutation on other cell types (astrocytes, neurons, oligodendrocytes). This idea derives from recent studies in which we found that neurons with a heterozygous *Nf1* mutation elaborate paracrine factors that act on T cells [21] and oligodendrocyte precursor cells [50] to modify their function.

To distinguish cell-autonomous from non-cell-autonomous microglia defects, we leveraged two different Cre driver lines to heterozygously delete the *Nf1* gene in either microglia or neural progenitor cells and their progeny (astrocytes, neurons, oligodendrocytes) in vivo. Surprisingly, we found that heterozygous *Nf1* loss in microglia had no effect on microglia function, whereas heterozygous *Nf1* loss in neural progenitor cells (and their progeny) recapitulated the sexually dimorphic microglial defects observed in *Nf1* ± mice.

## Results

### Proteomic analysis reveals differences in microglial protein expression between male WT and *Nf1* ± mice

**Fig. 1:**
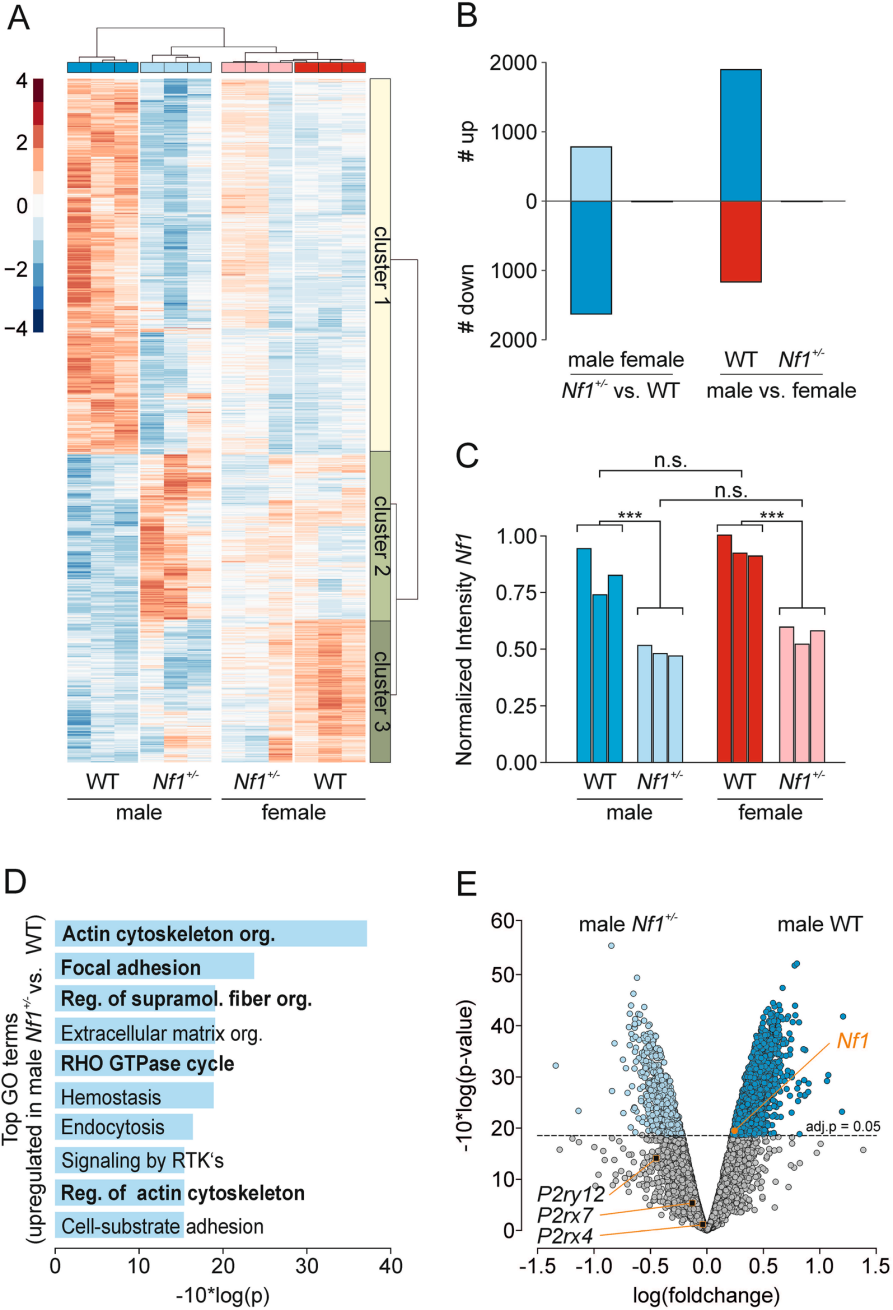
Proteomic analysis reveals sex-specific differences in WT and *Nf1* ± microglia **A:** Heatmap of significantly regulated proteins (adj. p < 0.05) in male and female WT and *Nf1* ± microglia. Cluster analysis revealed high in-group similarities, indicating that each sample did highly correlate to samples from the same group and less to those from other groups. Furthermore, differentially expressed genes clustered into 3 different major clusters which are annotated and analyzed in more detail in Additional File 1: Fig. S1.B: Number of significantly regulated proteins between the indicated pairwise comparisons. Note that there were many expressional differences between male WT and *Nf1* ± , as well as between male and female WT, microglia. Only a few significant differences were found between male *Nf1* ± vs. female *Nf1* ± microglia and female WT vs. female *Nf1* ± microglia. **C:** Comparison of *Nf1* protein levels in the analyzed samples. As expected, *Nf1* expression was reduced by ~ 50% in male and female *Nf1* ± compared to WT samples. **D:** GO term analysis of proteins upregulated in male *Nf1* ± compared to male WT microglia. Analysis was performed using Metascape [74]. Note that many terms refer to microglia morphology and surveillance. **E**: Volcano Plot comparing proteomic data from male WT and *Nf1* ± microglia. Position of *Nf1* is highlighted by an orange circle; those of the purinergic receptors *P2ry12*, *P2rx4* and *P2rx7* by black squares

To define *Nf1*-dependent alterations in murine microglia, we performed an unbiased proteomic analysis. Microglia were isolated from whole brains of male and female WT and *Nf1* ± mice (12- 16 weeks of age) by MACS using CD11b antibodies and subjected to proteomics analysis as previously described Mertins et al. [46] using TMTpro isobaric labeling. Hierarchical clustering of normalized intensities of all significantly differentially expressed proteins (adjusted p value: adj. p < 0.05) led to a clear separation into male and female, as well as WT and *Nf1* ± subclusters (Fig. 1A), indicating that differences in microglia protein expression occurred between, rather than within, groups. Clustering of differentially expressed genes revealed three major clusters, where cluster 2 and 3 were dominated by morphology- and membrane-related Gene Ontology (GO) networks and cluster 1 by transcriptional and mRNA-modifying terms (Additional file 1). Interestingly, among the data set there was a large number of differentially expressed proteins between male WT and *Nf1* ± microglia (2474), but none between female WT and *Nf1* ± microglia (Fig. 1B). As expected, and in accordance with our previous studies [20, 65], there were sex-dependent differences in the proteomes from WT microglia (3165 significantly regulated proteins), which were eliminated in the context of *Nf1* mutation (only five significantly regulated proteins observed). Consistent with a heterozygous *Nf1* mutation, there was a ~ 50% reduction in neurofibromin expression in *Nf1* ± relative to WT microglia (male: p = 0.0006; female: p = 0.0003; Fig. 1C).

Focusing more on male WT and *Nf1* ± microglia, we performed a GO analysis of the 789 proteins with increased expression in male *Nf1* ± versus WT microglia using Metascape [74]. Many of the GO terms were related to cytoskeletal organization (Fig. 1D), suggesting *Nf1*-dependent alterations in morphology or motility. Conversely, analysis of the downregulated proteins revealed GO terms that were associated with regulation of transcription and mRNA transport (Additional file 1: Fig. S2. There were three purinergic receptors found in our proteomic data set; *P2ry12*, *P2rx4* and *P2rx7*, and in accordance to data from our previous study, these proteins were not differentially expressed between WT and *Nf1* ± groups (Fig. 1E).

**Fig. 2:**
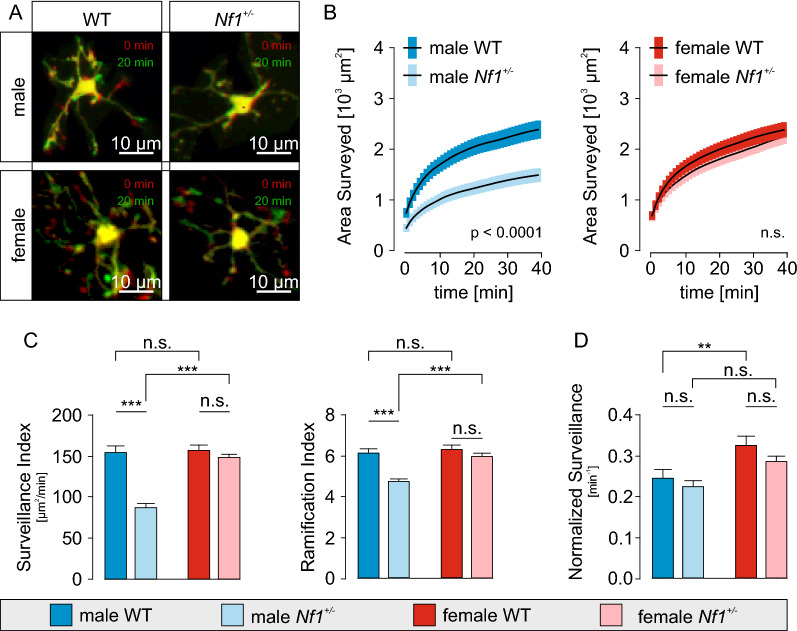
Microglia surveillance is reduced in male *Nf1* ± mice due to decreased ramification **A:** Sample images of male and female WT and *Nf1* ± microglia during 2-photon live cell recording. The MacGreen transgenic mouse lines express GFP in microglia and the cells can be visualized by fluorescence microscopy. Each image is an overlay of the time points t = 0 (red) and t = 20 min (green), thus, process extensions appear in green, retractions in red and resting parts in yellow. Scale bars denote 10 µm. **B:** Time course of increase of the number of surveyed pixels in maximum-intensity projections of images of male (*left*) and female (*right*) WT and *Nf1* ± microglia. The first value in the curves is equal to the area of the cell in the first image frame. **C:** Comparison of surveillance (*left*) and ramification (*right*) indices of microglia from male and female WT and *Nf1* ± microglia. **D:** Microglia surveillance normalized to cell area is an expression of the process motility independently of ramification. Note that the normalized surveillance was not different between male WT and male *Nf1* + */*

### Male *Nf1* ± microglia exhibit reduced surveillance and ramification

Based on the proteomic pathway findings, we next analyzed microglial properties related to cytoskeletal organization. First, we examined surveillance in acute cortical slices from 12–16 week old male and female mice, as previously reported Madry et al. [42]. The microglial cells in the slices were identified by transgenic EGFP labeling in WT and *Nf1* ± mice intercrossed with MacGreen (Csfr1-EGFP) mice. To avoid any bias toward microglia activation at the slice surface [24, 27], all experiments were performed on microglial cells located 50–100 μm below the surface of the slice within 4 h of acute slice preparation. Consistent with the proteomic results, the determined surveillance parameters of male *Nf1* ± microglia were reduced relative to their WT counterparts. The cumulative time courses of the surveyed area in maximum intensity projections (Fig. 2A and B) indicate that the initial rate of surveillance within 5 min was reduced in male *Nf1* ± microglia (74 ± 6 µm^2^/min; n = 106 cells/4 mice; p < 0.0001) relative to male WT microglia (129 ± 9 µm^2^/min; n = 53 cells/2 mice), and that the cumulative area surveyed after 40 min was also smaller in male *Nf1* ± (1488 ± 75 µm^2^; p < 0.0001) relative to male WT (2384 ± 117 µm^2^) microglia. Consequently, the surveillance index, which is a measure of the process retractions and extensions per unit time, was reduced in male *Nf1* ± microglia (86.4 ± 5.1 µm^2^/min) compared to male WT microglia (153.5 ± 8.7 µm^2^/min; p < 0.0001; Fig. 2C). Second, we determined the ramification index, which is a normalized parameter expressing the ratio of the cell perimeter to the perimeter of a perfect circle with the same area as the cell and, thus, depends only on cell shape, rather than its overall size [36]. As shown in Fig. 2C, male *Nf1* ± microglia had a ramification index (4.8 ± 0.1; n = 106 cells/4 mice) that was smaller than that observed for male WT microglia (6.2 ± 0.2; n = 53 cells/2 mice; p < 0.0001). The number of surveyed pixels and the surveillance index (Fig. 2B and C) might depend on the number and length of microglia processes or their speed of movement [36, 42]. We therefore normalized the surveillance indices to the mean area of each cell and found that there were no differences between male WT (0.25 ± 0.02 min^−1^) and male *Nf1* ± (0.23 ± 0.01 min^−1^, p = 0.3007) microglia (Fig. 2D). Based on these results, we conclude that the decreased surveillance of male *Nf1* ± microglia largely arises from their decreased ramification, rather than altered cell motility. Importantly, in female mouse brains, there were no differences in the surveillance of WT and *Nf1* ± microglia processes (Fig. 2A and B, neither in the initial rates of surveillance (0–5 min; female WT: 134 ± 10 µm^2^/min; n = 78 cells/3 mice; female *Nf1* ± : 108 ± 9 µm^2^/min; n = 95/3 mice; p = 0.0629) nor in the cumulative areas surveyed after 40 min (female WT: 2375 ± 90 µm^2^; female *Nf1* ± : 2242 ± 86 µm^2^; p = 0.2891). In addition, the surveillance indexes were similar in female WT (162.1 ± 6.5) and *Nf1* ± (146.7 ± 6.3; p = 0.0907) microglia. Taken together, these data demonstrate a sexually dimorphic reduction of ramification, resulting in reduced surveillance in *Nf1* ± microglia.

**Fig. 3:**
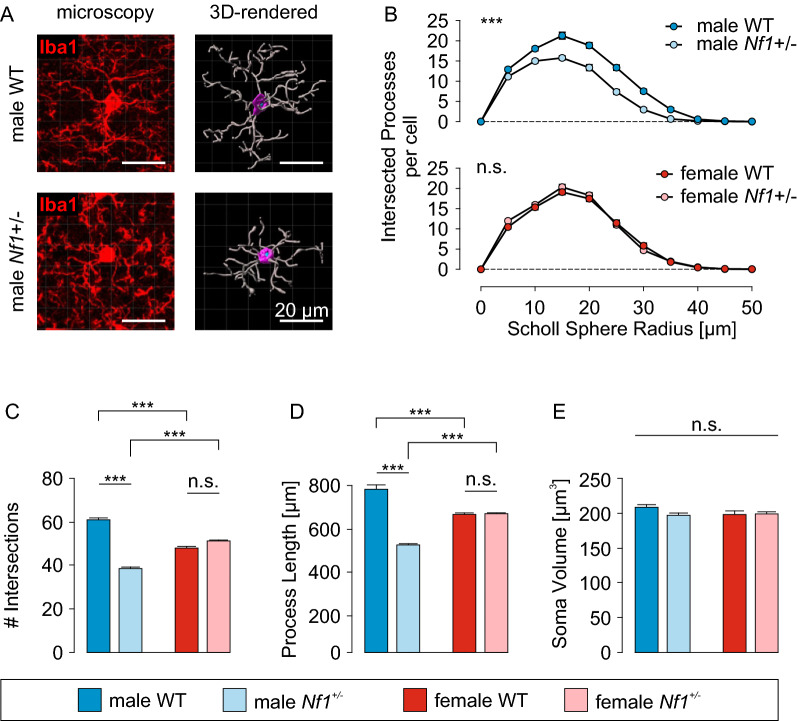
Microglia morphology is altered in male *Nf1* ± mice **A:** Representative confocal microscopic images (left) and 3-dimensional rendering (right) of a male WT and *Nf1* ± microglial cell in the cortex (layer 2–6). Scale bars denote 20 µm. **B:** Sholl analysis of male (*top*) and female (*bottom*) WT and *Nf1* ± microglia. The number of intersected processes was plotted against their distance from the soma. Male *Nf1* ± cells have a significantly reduced number of Sholl intersections in a radius of 5–40 μm around the soma compared to male WT microglia. **C:** Summary of the number of intersections of male and female WT and *Nf1* ± microglia. **D:** Summary of the total process length of male and female WT and *Nf1* ± microglia. **E:** Summary of the soma volumes of male and female WT and *Nf1* ± microglia. Number of quantified cells (mice): 50 (3) for each group

To explore microglia cytoskeleton-dependent processes in more detail, we analyzed microglial morphology in fixed male and female WT and *Nf1* ± mouse cortical brain slices (Fig. 3). Confocal images were taken with a 40X oil immersion objective on a Zeiss LSM700 inverse microscope at a resolution of 0.156 × 0.156 x 1 µm/voxel and an excitation wavelength of 639 nm. Three-dimensional rendering was applied using Imaris software (Fig. 3A) to perform 3D Scholl analysis and quantify the average soma volumes, total process lengths and number of branch points per cell. As observed with microglia surveillance and ramification, only microglia from male *Nf1* ± mice were less ramified relative to their WT counterparts, as indicated by the quantification of intersected processes within Scholl spheres at increasing distances from the soma (n = 50 cells from 3 mice per group; Fig. 3B). This result reflects reduced number of branch points per microglia in male *Nf1* ± (39 ± 1) compared to WT mice (61 ± 2; p < 0.0001; Fig. 3C), whereas female WT (48 ± 2) and female *Nf1* ± (51 ± 1; p = 0.3627) microglia had similar numbers. In addition, the cumulative process length per microglia was longer in male WT microglia (782 ± 20 μm) relative to male *Nf1* ± microglia (526 ± 14 μm; p < 0.0001; Fig. 3D), but no differences were observed between female WT (667 ± 17 μm) and female *Nf1* ± (670 ± 14 μm; p = 0.9988) microglia. In contrast to microglia process ramification, heterozygous *Nf1* loss had no impact on microglial soma volumes (Fig. 3E), which were similar in male and female WT microglia (209 ± 5 μm^3^ and 197 ± 7 μm^3^, respectively) and in *Nf1* ± male and female microglia (196 ± 5 μm^3^ and 198 ± 5 μm^3^, respectively). Collectively, these data provide additional support for a sex-by-genotype effect of *Nf1* heterozygosity on microglia process ramification.

### Microglia morphology and function is not altered by microglia-intrinsic heterozygous *Nf1* loss

**Fig. 4:**
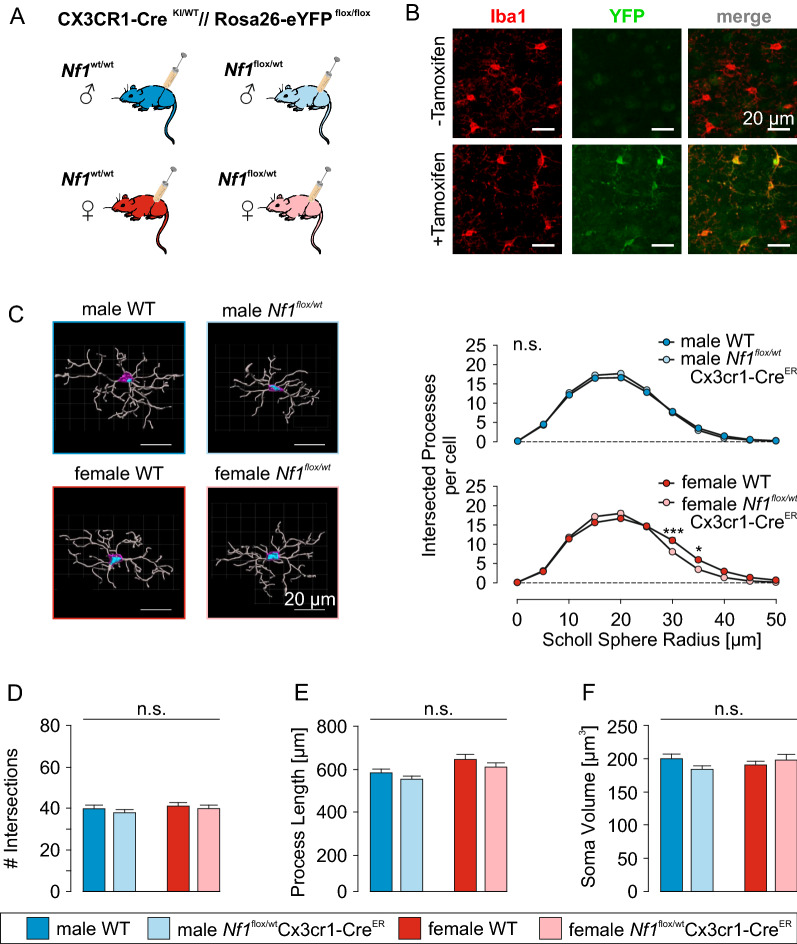
Microglia morphology is not altered by intrinsic *Nf1* reduction **A:** CX3CR1-Cre^ER^ mice were intercrossed with *Nf1*^flox/flox^ and R26R-EYFP to obtain litters that carry a heterozygous knock-in of Cre^ER^ at the CX3CR1 locus, a heterozygous knock-in of a flox cassette in the *Nf1* locus, and a homozygous, floxed eYFP transgene in the Rosa26 locus (*Nf1*^*flox/wt*^Cx3cr1-Cre^ER^). Tamoxifen was administered between P30 and P40 on five consecutive days to activate Cre recombinase in microglia. **B:** Confocal microscopic images indicating the successful induction of microglial YFP reporter expression by tamoxifen treatment in the cortex of two male WT mice. All scale bars are 20 µm. **C:** *Left,* 3-dimensional renderings of example male and female WT and *Nf1*^*flox/wt*^Cx3cr1-Cre^ER^ microglia in the somatosensory and parts of the motor cortex (layer 2–6). Scale bars denote 20 µm. *Right*, Sholl analysis of male (*top*) and female (*bottom*) WT and *Nf1*^*flox/wt*^Cx3cr1-Cre^ER^ microglia. The number of intersected processes was plotted against their distance from the soma. There was no difference in the distribution of process branches around the soma between the four investigated groups. **D-F:** Summary of the number of intersections per cell (D), the total process length (E) and the soma volumes (F) for male and female WT and *Nf1*^*flox/wt*^Cx3cr1-Cre^ER^ microglia. Number of quantified cells (mice): 50 (3) for each group

To determine whether the observed *Nf1* ± sexually dimorphic microglia alteration in ramification was a direct consequence of *Nf1* mutation on microglia biology (cell-intrinsic effect), we generated mice in which *Nf1* was heterozygously deleted in microglia (Fig. 4A). CX3CR1-Cre^ER^ [71] were intercrossed with *Nf1*^flox/flox^ [1] and R26R-EYFP mice [63] to generate litters heterozygous for Cre^ER^ expression from the CX3CR1 locus, a single conditional (flox) *Nf1* allele, and a conditional (LoxP-stop-LoxP; LSL) eYFP transgene in the Rosa26 locus (*Nf1*^flox/wt^; Cx3cr1-Cre^ER^; LSL-eYFP mice; termed *Nf1*^*flox/wt*^Cx3cr1-Cre^ER^ mice). Tamoxifen (100 mg/kg body weight) was intraperitoneally administered between P30 and P40 for five consecutive days to induce Cre recombinase activity in CX3CR1^+^ cells. WT (Cx3cr1-Cre) mice had the same genotype (*Nf1* + / + ; Cx3cr1-Cre^ER^; LSL-eGFP), except they lacked a conditional *Nf1* allele. As a control for the successful activation of Cre^ER^ in WT and *Nf1*^*flox/wt*^Cx3cr1-Cre^ER^ mice by tamoxifen treatment, cortical brain slices were immunostained with Iba1 and YFP antibodies. We observed a clear induction of microglial YFP expression in mice treated with tamoxifen at both neonatal and adult stages (Fig. 4B). There was also weak expression of YFP in non-microglial cells, mainly in neuronal cells independent of Cre^ER^ activation, as YFP was also seen in CX3CR1^wt/wt^ mice, which do not express Cre^ER^ recombinase (Additional file 1: FigS3). These unexpected findings likely reflect non-specific, off-target, expression of YFP from the Rosa26 locus, as has been previously reported for other reporter strains [73].

To determine the effect of heterozygous *Nf1* loss in microglia, cortical brain slices from male and female WT and *Nf1*^*flox/wt*^Cx3cr1-Cre^ER^ mice were analyzed (Fig. 4C). Confocal images were taken with a 40X oil immersion objective on a Leica SPE upright microscope at a resolution of 0.179 × 0.179 x 0.9 µm/voxel and an excitation wavelength of 635 nm. Three-dimensional rendering was applied using Imaris software to generate 3D quantification of microglia morphology. Interestingly, unlike microglia from *Nf1* ± mice, in which *Nf1* is heterozygously deleted in all cells, neither male nor female *Nf1*^*flox/wt*^Cx3cr1-Cre^ER^ microglia exhibited changes in process ramification relative to their WT counterparts (Fig. 4C). Additionally, there were similar numbers of branch points per microglia in male *Nf1*^*flox/wt*^Cx3cr1-Cre^ER^ (38 ± 2, n = 50) relative to male WT (40 ± 2; n = 50; p = 0.3670; Fig. 4D) microglia, which were also similar in female WT (40 ± 2; n = 50) and female *Nf1*^*flox/wt*^Cx3cr1-Cre^ER^ (38 ± 1; n = 50; ANOVA p = 0.8230) microglia. Likewise, quantification of intersected processes within Scholl spheres at increasing distances from the soma revealed a similar distribution in microglia from *Nf1*^*flox/wt*^Cx3cr1-Cre^ER^ and WT mice of either sex (Fig. 4C). There was no difference in the cumulative process length per microglial cell (male WT: 588 ± 17 μm; male *Nf1*^*flox/wt*^Cx3cr1-Cre^ER^: 567 ± 16 μm; female WT: 650 ± 23 μm; female *Nf1*^*flox/wt*^Cx3cr1-Cre^ER^: 603 ± 17 μm; n = 50 cells/3 mice per group; ANOVA p = 0.0711; Fig. 4E). There was also no effect of microglia-restricted heterozygous *Nf1* loss on microglia soma volumes (Fig. 4F), which were similar in male and female WT (194 ± 6 μm^3^ and 186 ± 5 μm^3^, respectively) and *Nf1*^*flox/wt*^Cx3cr1-Cre^ER^ (183 ± 5 μm^3^ and 187 ± 7 μm^3^, respectively; ANOVA p = 0.0502) mice. Collectively, these data demonstrate that microglia-specific heterozygous *Nf1* loss does not affect microglial morphology.

Second, to determine whether *Nf1* ± microglial functional defects were cell-autonomous, we measured microglial responses in acute brain slices of adult (12 -16 weeks) male and female WT and *Nf1*^*flox/wt*^Cx3cr1-Cre^ER^ mice following a laser lesion by two-photon imaging (Additional File 1: FigS4). The movement of microglial processes was quantified by determining the fluorescence distribution within the area of concentric circles (diameter: 20 µm and 90 µm) around the lesion site. In contrast to our previous findings using germline *Nf1* ± mice [17, 18], YFP-based fluorescence in mice was much weaker; however, detection of process movements was still possible (Additional File 1: Fig. S4A). No translocation of microglial cell bodies was seen during the observation period. In addition, similar to morphology (Fig. 4), there were no defects in directed process motility of microglia from *Nf1*^*flox/wt*^Cx3cr1-Cre^ER^ mice. The process response of male WT microglia 30 min following laser lesion was 24.6 ± 5.8 (n = 8), which was not different from that observed in male *Nf1*^*flox/wt*^Cx3cr1-Cre^ER^ (25.4 ± 4.0; n = 8; p = 0.9097). There were also no differences in process movements of female WT (27.4 ± 5.3; n = 8) relative to female *Nf1*^*flox/wt*^Cx3cr1-Cre^ER^ microglia (23.7 ± 5.2; n = 12; p = 0.6275).

Process movement in response to injury [17, 18, 20, 27, 42] is a P2RY12-mediated function in microglia [13, 48]. Microglial membrane current responses following the application of low (10 µM) ATP concentrations largely depend on P2RY12 activation [17, 18], which activates K^+^ currents through THIK-1 [3, 4, 20, 42, 67]. For these reasons, we analyzed purinergic responses of microglia in acute cortical slices from 12 to 16 weeks-old male and female *Nf1*^*flox/wt*^Cx3cr1-Cre^ER^ mice following tamoxifen administration at P30-40 using standard whole-cell patch clamp techniques in the voltage-clamp configuration. Microglial cells were identified in situ by eYFP fluorescence. WT and *Nf1*^*flox/wt*^Cx3cr1-Cre^ER^ microglial cells responded to 10 µM ATP with the induction of an outwardly rectifying current that reversed close to the equilibrium potential for potassium (Additional File 1: Fig. S4D-F). We next assessed the specific conductance between + 20 mV and + 60 mV to compare these currents in male and female microglia. The conductance of microglial ATP-evoked potassium currents was 13.5 ± 4.6 pS/pF and 13.6 ± 2.3 pS/pF for male and female WT microglia, respectively (n = 3 and n = 11 cells, respectively; p = 0.9823, Additional File 1: Fig. S3H). There were no differences in microglial purinergic responses in male *Nf1*^*flox/wt*^Cx3cr1-Cre^ER^ (15.0 ± 3.4 pS/pF; n = 14; p = 0.8550 vs. male WT) or female *Nf1*^*flox/wt*^Cx3cr1-Cre^ER^ (14.6 ± 3.1 pS/pF; n = 14; p = 0.8060 vs. female WT) mice, indicating that the microglia-specific heterozygous *Nf1* loss has no impact on P2RY-dependent membrane responses. Additionally, we compared the membrane characteristics of male and female WT and *Nf1*^*flox/wt*^Cx3cr1-Cre^ER^ microglia, and repetitively patch clamped cortical microglia at potentials between −170 and + 60 mV, starting from a holding potential of −70 mV. As shown in Additional File 1: Fig. S5, current density–voltage relations were characterized by a high input resistance and a small inwardly rectifying conductance between -40 and -170 mV, consistent with previous studies [33]. The reversal potentials, indicative of the resting membrane potential, were also similar among groups. There were no differences in the apparent membrane capacitance between the two sexes and genotypes. Taken together, these findings establish that the male microglia defects observed in *Nf1* ± mice were unlikely to be cell-autonomous.

### Microglia defects result from heterozygous *Nf1* loss in other brain cells

Based on the above findings, we considered the possibility that the sex by genotype effects reflected microglia responses to heterozygous *Nf1* mutation in other brain cells (e.g. neurons, astrocytes and oligodendrocytes). To address this question, we generated mice in which *Nf1* is heterozygously deleted in non-microglial cells (neural progenitor cells by embryonic day 16.5, hGFAP-Cre mice; by intercrossing *Nf1*^flox/flox^ and hGFAP-Cre mice [1]).

**Fig. 5:**
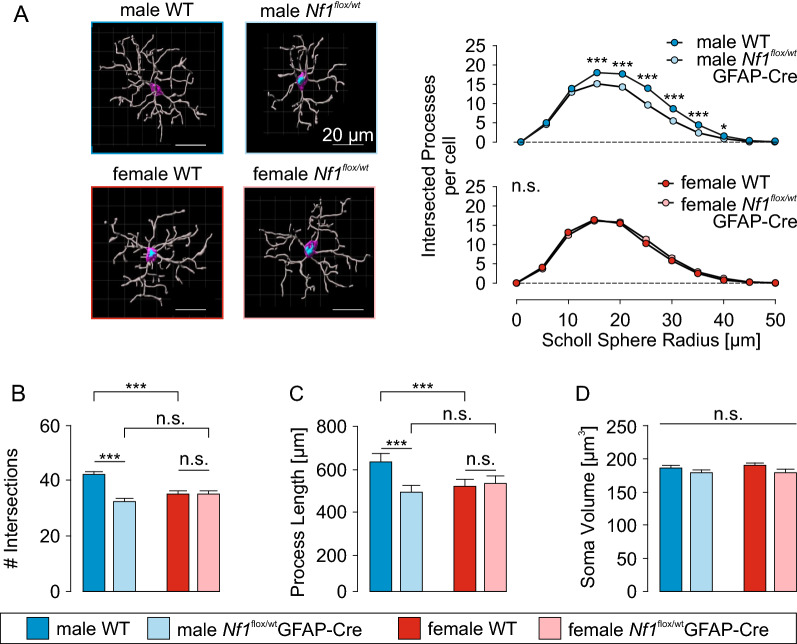
Microglia morphology is altered by extrinsic *Nf1* reduction **A:** *Left,* 3-dimensional renderings of example male and female WT and *Nf1*^*flox/wt*^; hGFAP-Cre microglia in the somatosensory and parts of the motor cortex (layer 2–6). Scale bars denote 20 µm. *Right*, Sholl analysis of male (*top*) and female (*bottom*) WT and *Nf1*^flox/wt^; hGFAP-Cre microglia. The number of intersected processes is plotted against their distance from the soma. There were significant differences in the intersected processes between the male WT and *Nf1*^*flox/wt*^ groups. **B-D:** Summary of the number of intersections per cell (D), the total process length per cell (E) and the soma volumes (F) for male and female WT and *Nf1*^*flox/wt*^; hGFAP-Cre microglia. Number of quantified cells (mice): 50 (3) for each group

To investigate microglia morphology, cells were analyzed as before, to quantify average soma volume, total process length and the number of branch points per cell (Fig. 5). Interestingly, microglia from male, but not female, *Nf1*^flox/wt^; hGFAP-Cre mice had decreased ramification. The quantification of intersected processes within Scholl spheres at increasing distances from the soma (n = 50 cells from 3 mice per group; Fig. 5A) revealed fewer intersections within a distance of 15–40 µm from the soma in male *Nf1*^flox/wt^; hGFAP-Cre, compared to WT microglia. In addition, the number of branch points per microglial cell was reduced in male *Nf1*^flox/wt^; hGFAP-Cre (32 ± 1) compared to WT microglia (42 ± 1; p < 0.0001; Fig. 5B), whereas female WT (35 ± 1) and female *Nf1* ± (35 ± 1; p > 0.9999) microglia were similar. Similar to *Nf1* ± mice (see Fig. 3), there was a sexually dimorphic decrease in the cumulative process lengths in male *Nf1*^flox/wt^; hGFAP-Cre microglia (500 ± 14 μm) relative to male WT microglia (652 ± 16 μm; p < 0.0001; Fig. 5C), and no differences were observed between female *Nf1*^flox/wt^; hGFAP-Cre (544 ± 16 μm) and female WT (530 ± 14 μm; p = 0.9018) microglia. In contrast to microglia process ramification, heterozygous *Nf1* loss in non-microglial cells had no impact on soma volume (Fig. 5D), which were similar in all groups (male WT: 185 ± 5 μm^3^, male *Nf1*^flox/wt^; hGFAP-Cre: 178 ± 5 μm^3^, female WT: 189 ± 5 μm^3^, female *Nf1*^flox/wt^; hGFAP-Cre: 178 ± 6 μm^3^, ANOVA p = 0.1818). Collectively, these findings indicate that the sex-by-genotype effects of *Nf1* reduction in microglia reflect the contributions of other cell types and are not cell-intrinsic to microglia.

## Discussion

In the present study, we report proteomic changes and defects in cortical microglia process ramification in male, but not female, *Nf1* ± mice. Using two different Cre driver lines, we demonstrate that the sexually dimorphic microglia defects observed in germline *Nf1* ± mice do not originate from heterozygous *Nf1* loss in microglia, but rather reflect responses to signals from sex- and *Nf1*-dependent alterations in other cell types in the brain. Leveraging mice in which heterozygous *Nf1* loss occurs in neural progenitor cells and their progeny (neurons, astrocytes and oligodendrocytes), but not in microglia, we show that heterozygous *Nf1* loss in the cellular environment mimics the sexually dimorphic microglia defects observed in germline *Nf1* ± mice. Taken together, these observations suggest that environmental factors from other brain cells induce microglia responses important for the formation and progression of NF1-related brain dysfunction.

Microglia are essential for the development and progression of numerous brain diseases [68], including neurodegenerative [72] and psychiatric disorders [44, 55], as well as brain tumors [23]; however, there is a great degree of heterogeneity between microglia associated with each brain disorder. In the early stages of Alzheimer’s disease, microglia facilitate the destruction of neuronal networks due to their ability to prune synapses [29, 29, 30, 30, 34], whereas the loss of phagocytic and surveillance activity contributes to enhanced Aß plaque burden at later stages [35, 67]. Similarly, schizophrenia is initiated and closely linked to inflammatory mechanisms [10], leading to a pro-inflammatory microglial phenotype [44, 45]. In high-grade (malignant) gliomas, microglia are transcriptionally reprogrammed by tumor-derived factors and act as tumor-supporting cellular elements [22, 23, 68], whereas in low-grade gliomas, microglia are essential for tumor growth in response to cytokines released by T lymphocytes [7, 14, 21, 51].

The findings in the current study exclude a cell-autonomous effect of heterozygous *Nf1* loss on microglia. As such, *Nf1* differs from other genes that directly affect microglia function through intrinsic mechanisms, such as *Cx3cr1* [52, 53], *P2ry12* [27, 61], *Gabbr1* [19], *Chrm3 *[11], *Apoe* [28], *Tgfbr2* [75], *Bmal1* [66], *Il6* [57]*, Il10* [31, 70] or *Bdnf* [47]. It should, however, be noted that all of these genes – in addition to their microglia-intrinsic effects—can also alter the behavior and function of non-microglial cells in a cell-autonomous fashion. Similarly, defects in neuronal *Bdnf* or *Bmal1* expression [26] [60] or macroglia cells [16] can influence microglia function. In contrast, microglia can also respond to genetic mutation in other cell types: whereas microglia in a mouse model of Rett’s syndrome (*Mecp2*-/-) excessively engulf, and thereby eliminate, presynaptic inputs at end stages of disease, conditional loss of *Mecp2* in microglia had little effect on synapse loss [59]. In addition, conditional loss of the calcitonin receptor (*Calcr*) in proopiomelanocortin (POMC) neurons of the hypothalamus leads to increased body weight gain, increased adiposity, and glucose intolerance [8] as a result of microglial *IL-6* release [32, 37]. However, *Calcr* loss in selective in microglia had no effect on body weight or glucose tolerance (Coester et al*.* 2022).

In the setting of *Nf1* heterozygosity, it is most likely that non-microglial cells (astrocytes, neurons or oligodendrocytes) are the initiating elements for the observed microglia morphological and functional changes. We acknowledge that it is conceivable that *Nf1* mutational effects on microglia might require heterozygous *Nf1* loss during embryonic development, prior to the Cre-mediated excision time point chosen for our experiments, which would require additional experimentation. Nonetheless, the simplest explanation for our findings suggest that normal microglia have the capacity to respond to paracrine or cell surface-bound cues provided by male *Nf1* ± neurons or macroglia. In keeping with this mechanism, we found that *Nf1* ± neurons secrete midkine, which can induce the expression of Ccl4 in both wild type and *Nf1* ± T cells (Guo et al*.* 2019). Further investigation will be required to determine whether both male and female wild type microglia can alter their biological properties in response to *Nf1* ± neurons or macroglia, or whether this sex by gene effect operates at the level of the neuron (or microglia). To this end, we have previously demonstrated sexually dimorphic differences between male and female *Nf1* ± neurons [15], which reflect sex by genotype effects on cyclic AMP generation and dopamine homeostasis. Additional studies will be necessary to determine whether differences in neuronal dopamine (or other paracrine factors) underlie the sexually dimorphic differences observed in microglia [2, 19, 39, 40, 64, 69]. Understanding how this intercellular crosstalk is established and maintained will provide new insights into our understanding of sex differences and the interplay between risk factors and cellular function in the brain.

## Materials and methods

### Ethics statement

All procedures involving the handling of living mice were performed in strict accordance with the German Animal Protection Law, and were approved by the Regional Office for Health and Social Services in Berlin (Landesamt für Gesundheit und Soziales, Berlin, Germany, Permit Number G0164/19, X9005/18, A0376/17). Adult mice were euthanized by cervical dislocation or by transcardial perfusion of PBS or PFA after intraperitoneal injection of pentobarbital (Narcoren, Merial GmbH, Hallbergmoos, Germany). All efforts were made to minimize suffering. Similarly, all mice used at Washington University were performed under an approved Animal Studies protocol.

### Mice

Wild type and *Nf1* ± [5] mice used in this study were either bred onto a wild type or MacGreen (Csf1r-eGFP; [58] C57/BL6 background. Conditional microglia-specific *Nf1* ± mice: CX3CR1-Cre^ER^ [71] were intercrossed with *Nf1*^flox/flox^ [1] and R26R-EYFP mice [63] to generate litters heterozygous for Cre^ER^ expression from the CX3CR1 locus, a conditional (flox) *Nf1* allele and a conditional (LoxP-stop-LoxP; LSL) eYFP transgene in the Rosa26 locus (*Nf1*^flox/wt^; Cx3cr1-Cre^ER^; LSL-eYFP mice; termed *Nf1*^flox/wt^CX3CR1-Cre^ER^ mice). Tamoxifen (100 mg/kg body weight) was administered intraperitoneally between days P30 and P40 on 5 consecutive days to include Cre recombinase activity in CX3CR1^+^ cells. Wild type (*Nf1*^wt/wt^; Cx3cr1-Cre^ER^; LSL-eYFP mice; termed WT) mice had the same genotype with the exception that they had no *Nf1*^flox^ allele. Conditional non-microglia-specific *Nf1* ± mice: hGFAP-Cre^+/+^ were intercrossed with *Nf1*^flox/flox^ [1] to generate litters heterozygous for Cre^ER^ expression from the GFAP locus and a conditional (flox) *Nf1* allele (*Nf1*^flox/wt^; hGFAP-Cre; termed *Nf1*^flox/wt^; hGFAP-Cre mice). Wild type (*Nf1*^wt/wt^; hGFAP-Cre; termed WT) mice had the same genotype with the exception that they had no *Nf1*^flox^ allele. All mice were maintained under a 12 h/12 h dark–light cycle with food and water supply ad libitum, in accordance with German laws and IACUC recommendations (U.S.A.) for animal protection. All data in the current study are from 12–16 week old mice. Sexes and genotypes are indicated.

### Immunohistochemistry and confocal microscopy

Mice were anesthetized with pentobarbital (Narcoren, Merial Hallbergmoos, Germany) and transcardially perfused with phosphate buffered salt solution (PBS) followed by 4% paraformaldehyde in PBS, decapitated and sectioned in the sagittal plane at 40 μm thickness using a sliding microtome (Leica SM2000 R, Leica Biosystems GmbH, Nussloch, Germany). Free-floating 40 μm sections were incubated in 5% donkey serum (EMD Millipore Corp., Burlington, Massachusetts, USA) and 0.1% Triton-X (Carl Roth®, Karlsruhe, Germany) in Tris-buffered saline solution (TBSplus) together with the primary antibodies over-night at 4 °C. The following primary antibodies were used: goat monoclonal Iba-1 antibody targeting microglia (1:300; Abcam, Cambridge, UK); chicken polyclonal GFP antibody targeting eYFP (1:250; Abcam, Cambridge, UK); mouse anti-NeuN targeting neurons (1:00; Synaptic Systems). After washing, secondary antibodies were prepared in TBSplus. Iba-1 was visualized with donkey anti-goat IgG conjugated with Cy5 or Alexa488 fluorophores (both 1:200; Dianova, Hamburg, Germany); eYFP was visualized with donkey anti-chicken IgY conjugated with Alexa488 or Cy5 (both 1:200; Dianova), NeuN with donkey anti-mouse IgG (H + L) conjugated with Cy3 (1:200; Dianova). Sections were incubated with secondary antibodies at room temperature for 2 h and then mounted on glass slides with Aqua Polymount mounting medium (Polysciences Europe GmbH, Hirschberg an der Bergstraße, Germany). Cell nuclei were stained using 4',6-diamidino-2-phenylindole (DAPI, 1:500; Dianova) before mounting. Images were acquired with either a Zeiss LSM700 (inverse) or a Leica SPE (upright; Leica Biosystems GmbH, Nussloch, Germany) using 40X oil immersion objectives. Z-stacks were taken at 1 μm z-step size, 35 steps to cover the whole thickness of the slice.

### Morphological analysis

Morphological analysis of microglia was performed on 3-dimensional fluorescence images using Imaris × 64 version 9.6–9.9 (Bitplane, Zurich, Switzerland) algorithms. Microglial cells in which the nucleus was at least 15 µm away from the image border were selected for analysis. The modules "Filament tracer" and "Surface" were used for microglia reconstruction. A total of 50 cells from 3 different mice were analyzed for each group. The background was minimized with an appropriate filter width (20–40 µm) and the region of the analyzed cell was selected manually. The parameters Filament Length, Filament No, Dendrite Branch Pts, Filament No, Sholl Intersections, and Soma Volume were obtained from the specific values calculated by Imaris. Although tracing was performed automatically by the algorithm, we individually verified that processes originated from one defined cell. False connections were removed manually which were commonly less than 1%. The number of Sholl intersections was defined as the number of process intersecting concentric spheres, defining the spatial distribution of segments as a function of distance from the soma (Sholl analysis). All spheres have their center at the soma (beginning point) with a 5 µm step resolution for the spheres.

### Acute brain slice preparation

Acute cortical brain slices were prepared as previously described [4]. In brief, mice were euthanized by cervical dislocation, and their brains removed and cooled in ice-cold artificial cerebrospinal fluid (aCSF) containing (in mM): 230 Sucrose, 2.5 KCl, 10 MgSO_4_, 0.5 CaCl_2_, 1.25 NaH_2_PO_4_, 26 NaHCO_3_, and 10 D-glucose, pH 7.4; gassed with 95% O_2_/ 5% CO_2_. Brains were then mounted on a vibratome (HM650V, Thermo Scientific, Massachusetts, USA), and 250 µm thick coronal brain slices were generated and kept at room temperature for experiments for up to 5 h in gassed ACSF containing (in mM): 134 NaCl, 2.5 KCl, 1.3 MgCl_2_, 2 CaCl_2_, 1.26 K_2_HPO_4_, 26 NaHCO_3_, and 10 D-glucose (pH 7.4). Acute brain slices were used for patch clamp recordings and in situ 2-photon live-cell imaging.

### Two-photon imaging and laser lesioning

Live imaging of microglial processes was performed on 250 µm coronal brain slices from *Nf1* ± or *Nf1*flox/wt mice and their WT littermates using a custom-built two-photon laser-scanning microscope (Till Photonics, Gräfelfing, Germany). EGFP or eYFP was excited by a Chameleon Ultra II laser (Coherent, Dieburg, Germany) at a wavelength of 940 nm. A 40X water-immersion objective (NA 0.8, Olympus, Hamburg, Germany) was used, with scanned 60 µm thick z-stacks and a step size of 3 µm covering a field of 320 × 320 µm. Laser lesions were set to 40 µm under the slice surface in the cortex by focusing the laser beam, set to a wavelength of 810 nm and to maximum power in the selected imaging volume, and scanned until autofluorescence of the injured tissue was visible. This procedure resulted in lesions of ~ 20 µm in diameter in the middle of the observed region. For the recording of microglia surveillance, no laser lesion was performed. IGOR Pro 6.37 (Lake Oswego, USA) was used for data analysis as in Davalos et al. [13] and Madry et al. [42]. The sequences of 3D image stacks were converted into sequences of 2D images by a maximum intensity projection algorithm. Grayscale images were first converted into binary form using a threshold. For quantification of laser lesion-induced movements, microglial response to focal lesion was defined as EGFP + pixel count in a proximal circular region 45 µm around the lesion site over time (Rx(t)). Distal fluorescence of the first time point was determined within a diameter of 45 µm to 90 µm around the lesion site for normalization (Ry(0)). Microglial responses were represented as R(t) = (Rx(t)-Rx(0))/Ry(0). For the quantification of baseline surveillance, cells of interest were individually selected by manually drawing a region of interest (ROI) around an area including all their process extensions throughout the 20 min movie and erasing data around that ROI. Starting with the second frame, we subtracted from each binarized frame the preceding frame and counted the number of pixels < 0 (retracting = PR) and > 0 (extending = PE). The surveillance index for each frame is then given by the sum of PR ad PE. The surveillance index of a given cell was then calculated by averaging the indices of the first 20 images in the movie. For ramification index (RI), we used the equation RI = (peri/area)/(2*sqrt(pi/area)), where peri and area are respectively the perimeter and area of a given cell in pixels. For the quantification of these two parameters, the ImageAnalyzeParticles operation in IGOR Pro 6.37 was applied on binarized images in which all analyzed microglia were manually examined and, if necessary, somata and processes connected.

### Electrophysiological recordings

A conventional patch-clamp amplifier was used (EPC9, HEKA Elektronik, Lambrecht, Germany). Acute 250 µm coronal brain slices were prepared from *Nf1*^flox/wt^ and WT mice, and microglial cells were identified by their transgenic EYFP fluorescence on an epifluorescent microscope. Patch pipettes were pulled from borosilicate glasses and had resistances of 4—6 MOhm. The following intracellular solution was used (in mM): KCl, 130; MgCl_2_, 2; CaCl_2_, 0.5; Na-ATP, 2; EGTA, 5; HEPES, 10 and sulforhodamine 101, 0.01 (Sigma Aldrich,) and had an osmolarity of 280—290 mOsm/L adjusted to a pH of 7.3 with KOH. The extracellular solution contained (in mM): NaCl, 134; KCl, 2.5; MgCl_2_, 1.3; CaCl_2_, 2; K_2_HPO_4_, 1.25; NaHCO_3_, 26; D-glucose, 10; pH 7.4; 310—320 mOsm/L and was gassed with carbogen (95% O_2_/ 5% CO_2_). Experiments with series resistances less than ~ 65 MOhm were used for data analysis. All experiments were performed in the voltage-clamp configuration. To obtain current–voltage curves during continuous recordings, the membrane was clamped every 5 s from a holding potential of -70 or -20 mV (before and during the ATP response, respectively) to a series of de- and hyperpolarizing voltages ranging from -140 mV to 60 mV with 20 mV increment, 100 ms in duration. Membrane currents were averaged for quantification between 30 and 45 ms after clamping the membrane to a given value from the resting potential. Membrane capacitance was quantified based on an exponential fit of the current decay in response to a -10 mV test pulse. The same pulse was used to quantify series resistance from the peak amplitude of the membrane capacitance currents. Comparisons of membrane currents between different groups were always normalized to the membrane capacitance.

### Microglia isolation by MACS

12–16 week male and female mice were transcardially perfused with ice-cold Phosphate Buffered Saline (PBS) to harvest the brain. Brains were subsequently homogenized at 0–4 °C in dissection buffer (HBSS, 45% glucose, 1 M HEPES) and cell pellets were resuspended in 25 ml of 22% Percoll (GE Healthcare, Little Chalfont, UK). 5 ml PBS were added as a layer on top. Centrifugation was performed for 20 min at 950 g with medium acceleration and no brakes to remove myelin and debris. Pellets were resuspended in ice-cold MACS buffer and incubated with anti-mouse CD11b antibodies coupled to magnetic beads (Miltenyi Biotech, Bergisch Gladbach, Germany) for 15 min at 4 °C. Cells were resuspended in MACS buffer and passed through medium-sized MACS columns (Miltenyi Biotech) attached to a magnet. The flow-through was discarded and the cells were flushed out of the columns in MACS buffer, collected by centrifugation, and stored at −80 °C for cAMP ELISA.

### Mass spectrometry and proteomic analysis

Global proteome analysis was conducted using isobaric TMTpro 16-plex labeling (Thermo Fisher Scientific) essentially as described in Mertins et al. [46]. Samples were lysed in urea buffer and digested with trypsin (Promega). Of each sample, 12 µg peptide was assigned to channels 1 through 13 as well as channel 15 in a randomized fashion. Channel 16 was used as a booster channel and loaded with 70 µg of a mix of the remaining peptide material. Channel 14 was kept empty. The combined TMT cassette was deeply fractionated using high-pH HPLC separation into 30 fractions using a 1290 Infinity II LC System (Agilent Technologies). An estimate of 1 µg of each fraction was injected into LC–MS analysis on an Orbitrap Exploris 480 mass spectrometer (Thermo Fisher Scientific) in data-dependent mode using a 110 min gradient on an EASY-nLC 1200 system (Thermo Fisher Scientific) with an in-house packed column (C18-AQ 1.9 µm beads,Dr. Maisch Reprosil-Pur 120). For database search, MaxQuant version 1.6.10.43 [12] was used whilst enabling TMTpro 16-plex reporter ion quantitation with a PIF setting of 0.5. Downstream analysis was done in R. For quantitation, a complete data matrix was required for the experimental conditions (14 out of 16 channels). Reporter ion intensities were normalized and scaled using median-MAD normalization.. For significance calling two-sample moderated t-tests were applied (limma R package; [56]. P-values were adjusted using the Benjamini–Hochberg method.

### Statistics

All data are expressed as mean ± SEM. A combination of one-way ANOVA tests with Bonferroni post hoc tests were employed using Prism 7 (GraphPad Software, San Diego, CA, USA) to compare data between the four experimental groups. Significance is given as *** p < 0.001, ** p < 0.01, * p < 0.05, n.s. p > 0.05.

## Supplementary Information

**Additional file 1:** Supplementary Figures.

## Data Availability

The mass spectrometry proteomics data have been deposited to the ProteomeXchange Consortium (http://proteomecentral.proteomexchange.org) via the PRIDE partner repository [54] with the dataset identifier PXD035881.

## References

[1] Bajenaru ML (2002). Astrocyte-specific inactivation of the neurofibromatosis 1 gene (NF1) is insufficient for astrocytoma formation. Mol Cell Biol.

[2] Bernier LP (2019). Nanoscale Surveillance of the Brain by Microglia via cAMP-Regulated Filopodia. Cell Rep.

[3] Boucsein C (2000). Electrophysiological properties of microglial cells in normal and pathologic rat brain slices. Eur J Neurosci.

[4] Boucsein C (2003). Purinergic receptors on microglial cells: functional expression in acute brain slices and modulation of microglial activation in vitro. Eur J Neurosci.

[5] Brannan CI (1994). Targeted disruption of the neurofibromatosis type-1 gene leads to developmental abnormalities in heart and various neural crest-derived tissues. Genes Dev.

[6] Cadiz MP (2022). Culture shock: microglial heterogeneity, activation, and disrupted single-cell microglial networks in vitro. Mol Neurodegener.

[7] Chatterjee J (2021). Asthma reduces glioma formation by T cell decorin-mediated inhibition of microglia. Nat Commun.

[8] Coester B (2020). Amylin/Calcitonin Receptor-Mediated Signaling in POMC Neurons Influences Energy Balance and Locomotor Activity in Chow-Fed Male Mice. Diabetes.

[9] Coester, B., et al. (2022). "Mouse Microglial Calcitonin Receptor Knockout Impairs Hypothalamic Amylin Neuronal pSTAT3 Signaling but Lacks Major Metabolic Consequences." Metabolites **12**(1). 10.3390/metabo12010051 PMC8780059 35050175

[10] Comer, A. L., et al. (2020). "The Inflamed Brain in Schizophrenia: The Convergence of Genetic and Environmental Risk Factors That Lead to Uncontrolled Neuroinflammation." Front Cell Neurosci **14**. 10.3389/fncel.2020.00274 PMC7518314 33061891

[11] Costa A (2021). Deletion of muscarinic acetylcholine receptor 3 in microglia impacts brain ischemic injury. Brain Behav Immun.

[12] Cox J, Mann M (2008). MaxQuant enables high peptide identification rates, individualized p.p.b.-range mass accuracies and proteome-wide protein quantification. Nat Biotechnol.

[13] Davalos D (2005). ATP mediates rapid microglial response to local brain injury in vivo. Nat Neurosci.

[14] de Andrade Costa, A., et al. (2022). "Immune deconvolution and temporal mapping identifies stromal targets and developmental intervals for abrogating murine low-grade optic glioma formation." Neurooncol Adv **4**(1): vdab194. 10.1093/noajnl/vdab194 PMC8852255 35187488

[15] Diggs-Andrews KA (2014). Sex Is a major determinant of neuronal dysfunction in neurofibromatosis type 1. Ann Neurol.

[16] Djalali S (2005). Effects of brain-derived neurotrophic factor (BDNF) on glial cells and serotonergic neurones during development. J Neurochem.

[17] Elmadany, N., et al. (2020). "The VGF-derived peptide TLQP21 impairs purinergic control of chemotaxis and phagocytosis in mouse microglia." J Neurosci. 10.1523/JNEUROSCI.1458-19.2020 PMC7178903 32060170

[18] Elmadany N (2020). Neurofibromatosis 1 - Mutant microglia exhibit sexually-dimorphic cyclic AMP-dependent purinergic defects. Neurobiol Dis.

[19] Favuzzi, E., et al. (2021). "GABA-receptive microglia selectively sculpt developing inhibitory circuits." Cell **184**(15): 4048–4063 e4032. 10.1016/j.cell.2021.06.018 PMC9122259 34233165

[20] Guneykaya D (2018). Transcriptional and Translational Differences of Microglia from Male and Female Brains. Cell Rep.

[21] Guo X (2020). Midkine activation of CD8(+) T cells establishes a neuron-immune-cancer axis responsible for low-grade glioma growth. Nat Commun.

[22] Gutmann DH, Kettenmann H (2019). Microglia/Brain Macrophages as Central Drivers of Brain Tumor Pathobiology. Neuron.

[23] Hambardzumyan D (2016). The role of microglia and macrophages in glioma maintenance and progression. Nat Neurosci.

[24] Hanisch UK, Kettenmann H (2007). Microglia: active sensor and versatile effector cells in the normal and pathologic brain. Nat Neurosci.

[25] Harrison JK (1998). Role for neuronally derived fractalkine in mediating interactions between neurons and CX3CR1-expressing microglia. Proc Natl Acad Sci U S A.

[26] Hastings MH (2020). Molecular-genetic Manipulation of the Suprachiasmatic Nucleus Circadian Clock. J Mol Biol.

[27] Haynes SE (2006). The P2Y12 receptor regulates microglial activation by extracellular nucleotides. Nat Neurosci.

[28] Henningfield CM (2022). Microglia-specific ApoE knock-out does not alter Alzheimer's disease plaque pathogenesis or gene expression. Glia.

[29] Hong S (2016). Complement and microglia mediate early synapse loss in Alzheimer mouse models. Science.

[30] Hong S (2016). New insights on the role of microglia in synaptic pruning in health and disease. Curr Opin Neurobiol.

[31] Huo S (2021). Upregulation of TRPC5 in hippocampal excitatory synapses improves memory impairment associated with neuroinflammation in microglia knockout IL-10 mice. J Neuroinflammation.

[32] Johnson MD (2016). Early postnatal amylin treatment enhances hypothalamic leptin signaling and neural development in the selectively bred diet-induced obese rat. Am J Physiol Regul Integr Comp Physiol.

[33] Kettenmann H (2011). Physiology of microglia. Physiol Rev.

[34] Kettenmann H (2013). Microglia: new roles for the synaptic stripper. Neuron.

[35] Krabbe G (2013). Functional impairment of microglia coincides with Beta-amyloid deposition in mice with Alzheimer-like pathology. PLoS ONE.

[36] Kyrargyri V (2020). P2Y13 receptors regulate microglial morphology, surveillance, and resting levels of interleukin 1beta release. Glia.

[37] Le Foll C (2015). Amylin-induced central IL-6 production enhances ventromedial hypothalamic leptin signaling. Diabetes.

[38] Lehrman EK (2018). CD47 Protects Synapses from Excess Microglia-Mediated Pruning during Development. Neuron.

[39] Liu YU (2019). Neuronal network activity controls microglial process surveillance in awake mice via norepinephrine signaling. Nat Neurosci.

[40] Logiacco F (2021). Microglia sense neuronal activity via GABA in the early postnatal hippocampus. Cell Rep.

[41] Machlovi SI (2022). APOE4 confers transcriptomic and functional alterations to primary mouse microglia. Neurobiol Dis.

[42] Madry C (2018). Microglial Ramification, Surveillance, and Interleukin-1beta Release Are Regulated by the Two-Pore Domain K(+) Channel THIK-1. Neuron.

[43] Marsh SE (2022). Dissection of artifactual and confounding glial signatures by single-cell sequencing of mouse and human brain. Nat Neurosci.

[44] Mattei D (2014). Minocycline rescues decrease in neurogenesis, increase in microglia cytokines and deficits in sensorimotor gating in an animal model of schizophrenia. Brain Behav Immun.

[45] Mattei D (2017). Maternal immune activation results in complex microglial transcriptome signature in the adult offspring that is reversed by minocycline treatment. Transl Psychiatry.

[46] Mertins P (2018). Reproducible workflow for multiplexed deep-scale proteome and phosphoproteome analysis of tumor tissues by liquid chromatography-mass spectrometry. Nat Protoc.

[47] Moy JK (2019). Temporal and sex differences in the role of BDNF/TrkB signaling in hyperalgesic priming in mice and rats. Neurobiol Pain.

[48] Nimmerjahn A (2005). Resting microglial cells are highly dynamic surveillants of brain parenchyma in vivo. Science.

[49] Ocañas, S. R., et al. (2022). "Minimizing the Ex Vivo Confounds of Cell-Isolation Techniques on Transcriptomic and Translatomic Profiles of Purified Microglia." eNeuro **9**(2). 10.1523/ENEURO.0348-21.2022 PMC8970438 35228310

[50] Pan Y (2021). NF1 mutation drives neuronal activity-dependent initiation of optic glioma. Nature.

[51] Pan Y (2018). Athymic mice reveal a requirement for T-cell-microglia interactions in establishing a microenvironment supportive of Nf1 low-grade glioma growth. Genes Dev.

[52] Paolicelli RC (2011). Synaptic pruning by microglia is necessary for normal brain development. Science.

[53] Parkhurst CN (2013). Microglia promote learning-dependent synapse formation through brain-derived neurotrophic factor. Cell.

[54] Perez-Riverol Y (2022). The PRIDE database resources in 2022: a hub for mass spectrometry-based proteomics evidences. Nucleic Acids Res.

[55] Petrelli F (2016). Astrocytes and Microglia and Their Potential Link with Autism Spectrum Disorders. Front Cell Neurosci.

[56] Ritchie ME (2015). limma powers differential expression analyses for RNA-sequencing and microarray studies. Nucleic Acids Res.

[57] Sanchis P (2020). Microglial cell-derived interleukin-6 influences behavior and inflammatory response in the brain following traumatic brain injury. Glia.

[58] Sasmono RT (2003). A macrophage colony-stimulating factor receptor-green fluorescent protein transgene is expressed throughout the mononuclear phagocyte system of the mouse. Blood.

[59] Schafer, D. P., et al. (2016). "Microglia contribute to circuit defects in Mecp2 null mice independent of microglia-specific loss of Mecp2 expression." Elife **5**. 10.7554/eLife.15224 PMC4961457 27458802

[60] Singh B (2006). Altered balance of glutamatergic/GABAergic synaptic input and associated changes in dendrite morphology after BDNF expression in BDNF-deficient hippocampal neurons. J Neurosci.

[61] Sipe GO (2016). Microglial P2Y12 is necessary for synaptic plasticity in mouse visual cortex. Nat Commun.

[62] Solga AC (2015). RNA-sequencing reveals oligodendrocyte and neuronal transcripts in microglia relevant to central nervous system disease. Glia.

[63] Srinivas S (2001). Cre reporter strains produced by targeted insertion of EYFP and ECFP into the ROSA26 locus. BMC Dev Biol.

[64] Stowell RD (2019). Noradrenergic signaling in the wakeful state inhibits microglial surveillance and synaptic plasticity in the mouse visual cortex. Nat Neurosci.

[65] Villa A (2018). Sex-Specific Features of Microglia from Adult Mice. Cell Rep.

[66] Wang XL (2021). Microglia-specific knock-down of Bmal1 improves memory and protects mice from high fat diet-induced obesity. Mol Psychiatry.

[67] Wendt S (2017). Changes in phagocytosis and potassium channel activity in microglia of 5xFAD mice indicate alterations in purinergic signaling in a mouse model of Alzheimer's disease. Neurobiol Aging.

[68] Wolf SA (2017). Microglia in Physiology and Disease. Annu Rev Physiol.

[69] Xia P (2021). Histamine triggers microglial responses indirectly via astrocytes and purinergic signaling. Glia.

[70] Yang L (2021). Depression-like behavior associated with E/I imbalance of mPFC and amygdala without TRPC channels in mice of knockout IL-10 from microglia. Brain Behav Immun.

[71] Yona S (2013). Fate mapping reveals origins and dynamics of monocytes and tissue macrophages under homeostasis. Immunity.

[72] Zhang, G., et al. (2021). "Microglia in Alzheimer’s Disease: A Target for Therapeutic Intervention." Front Cell Neurosci **15**. 10.3389/fncel.2021.749587 PMC8651709 34899188

[73] Zhao, X. F., et al. (2019). "Targeting Microglia Using Cx3cr1-Cre Lines: Revisiting the Specificity." eNeuro **6**(4). 10.1523/ENEURO.0114-19.2019 PMC6620394 31201215

[74] Zhou Y (2019). Metascape provides a biologist-oriented resource for the analysis of systems-level datasets. Nat Commun.

[75] Zöller T (2018). Silencing of TGFβ signalling in microglia results in impaired homeostasis. Nat Commun.

