# Supplementary material for: Neurofibromatosis type 1-dependent alterations in mouse microglia function are not cell-intrinsic
Source: Acta Neuropathol Commun. 2023 Mar 9;11:36. doi: 10.1186/s40478-023-01525-w (PMC9996880; doi:10.1186/s40478-023-01525-w)

**Supplementary Figure 1.** GO networks of Clusters 1-3

GO network analysis was performed by Metascape (Zhou *et al.* 2019) and revealed that differentially expressed proteins in Cluster 1 are mainly described by biological terms connected to DNA- and mRNA-connected processes whereas biological terms in Clusters 2 and 3 are related to membrane processes and morphological features.

**Supplementary Figure 2.** Additional proteomic data

**A:** PCA plot of proteomic data from male and female WT and *Nf1+/-* samples. Only significantly regulated proteins (adj. p < 0.05) were used to generate the plot. Note that samples from the same group cluster together and that WT groups are more homogeneous than *Nf1+/-*.

**B:** GO term analysis of proteins downregulated in male *Nf1+/-* compared to male WT microglia. Analysis was performed using Metascape (Zhou *et al.* 2019).

**C**: Volcano Plots comparing proteomic data from male and female WT (left), male and female *Nf1+/-* (middle) and female WT and female *Nf1+/-* microglia (right).

**Supplementary Figure 3.** YFP reporter expression in CX3CR1-Cre^ER^ mice

Confocal images of cortical brain slices from LSL-eYFP mice (Rosa26-YFP^flox/flox^) without (top), with a heterozygous (middle) or a homozygous knock-in of Cre^ER^ at the *Cx3cr1* locus. Slices were immunohistochemically stained against YFP (green) and Iba1 (red). Mice were not treated with tamoxifen. Note the weak expression of YFP in non-microglial cells indicating a Cre-independent leaky expression of the YFP transgene.

**Supplementary Figure 4.** Lesion-induced process movement and ATP evoked currents are not altered in *Nf1^flox/wt^;* Cx3Cr1-Cre^ER^ microglia.

**A:** Representative pictures of injury-induced process movement of eYFP-positive microglia in the cortex of a male *Nf1^flox/wt^;* Cx3Cr1-Cre^ER^ mouse. Acute injury was induced by a focal laser lesion (*top*) and microglial movement activity was recorded for 30 min (*bottom*). Yellow lines indicate the concentric circles (solid: 20 µm; dotted: 90 µm) around the lesion site used for offline quantification of microglial process movements.

**B, C:** Quantitative analysis of lesion-induced, microglial process movement expressed in arbitrary units (AU) in male and female WT and *Nf1^flox/wt^;* Cx3Cr1-Cre^ER^ mice.

**D:** Representative patch-clamp recording from a cortical male *Nf1^flox/wt^;* Cx3Cr1-Cre^ER^ microglia. *Top*, From a holding potential of ‑20 mV, cells were repetitively clamped at a series of potentials between -140 and +60 mV every 5 s. Application of 10 µM ATP is indicated by the bar. Note the typical metabotropic purinergic response to 10 µM ATP with activation of outwardly rectifying currents. *Bottom*, Current-voltage relationships of currents at the indicated time points (1 and 2) in the respective time courses on the top. P2Y-mediated current responses (light blue) were obtained by subtraction of microglial membrane currents prior to and in the presence of 10 µM ATP.

**E, F:** Averaged current density-voltage relationships of currents evoked by 10 µM ATP in male (**F**) and female (**G**) WT and *Nf1^flox/wt^;* Cx3Cr1-Cre^ER^ microglia. Current responses were obtained by subtraction of microglial membrane currents prior to and in the presence of 10 µM ATP, followed by normalization to the capacitance.

**G:** Quantitative analysis of laser lesion-induced, microglial process movement expressed in arbitrary units (AU). Data refer to the experiments shown in **A-C**.

**H:** Summary of the specific outward conductances of ATP-evoked K^+^ currents. There was no significant difference throughout the investigated groups. Data refer to the experiments shown in **D-F**.

**Supplementary Figure 5.** Membrane properties of *Nf1^flox/wt^;* Cx3Cr1-Cre^ER^ microglia are similar to WT microglia

**A:** Sample images taken with the epifluorescence microscope during patch clamp recordings. Animals were tamoxifen-treated between P30 and P40. Left: male WT, Right: male *Nf1^flox/wt^;* Cx3Cr1-Cre^ER^. The large image was taken with a 5X objective and shows the investigated cortical region. The two smaller images were taken with a 63X objective and transmission (top) or fluorescence (bottom) illumination.

**B:** Sample currents of microglia from 12-16 week old WT and *Nf1^flox/wt^;* Cx3Cr1-Cre^ER^mice, which were obtained during a series of voltage pulses ranging from ‑170 to +60 mV, with 10 mV increments from a holding potential of ‑70 mV.

**C, D:** Summarized and averaged current-voltage relationships from 12-16 week old female (C) or male (D) WT and *Nf1^flox/wt^;* Cx3Cr1-Cre^ER^ microglia. Currents were normalized to the membrane capacitance before averaging. See panel A for sample traces. There were no significant sex- or *Nf1*-dependent differences (ANOVA/Tukey).

**E**: Distribution of the reversal potentials (indicative of the membrane potential), shown as averaged histograms of all recorded microglial cells from male and female WT and *Nf1^flox/wt^;* Cx3Cr1-Cre^ER^ mice.

**F**: Summary of the membrane capacitances of microglia from WT and *Nf1^flox/wt^;* Cx3Cr1-Cre^ER^ mice. There were no significant sex- or *Nf1*-dependent differences (ANOVA/Tukey).

Number of recorded cells (mice): male WT, 3 (1); male *Nf1^flox/wt^;* Cx3Cr1-Cre^ER^, 13 (3); female WT, 10 (2); female *Nf1^flox/wt^;* Cx3Cr1-Cre^ER^, 11 (4).


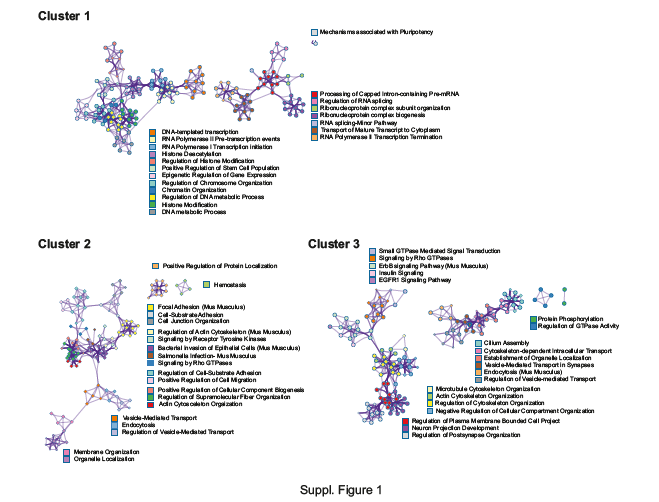


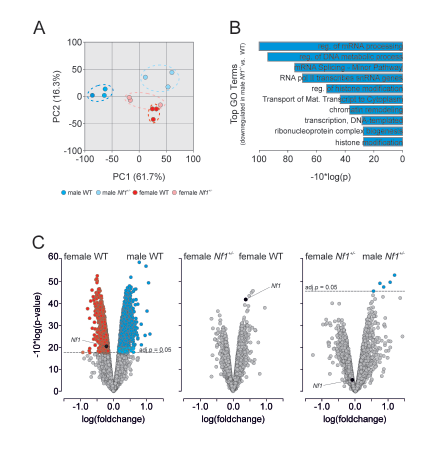


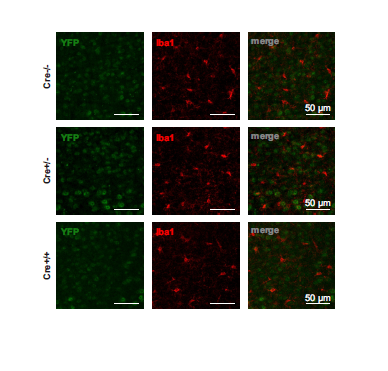

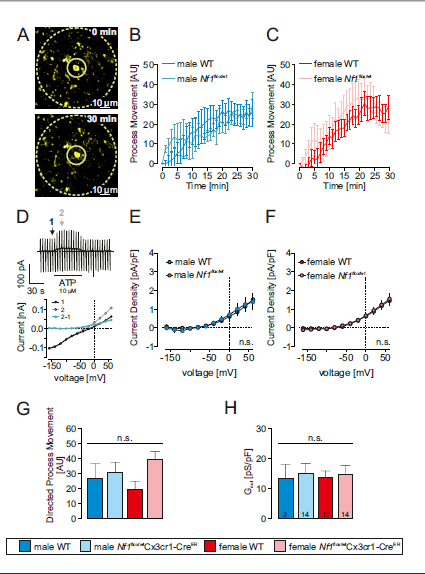


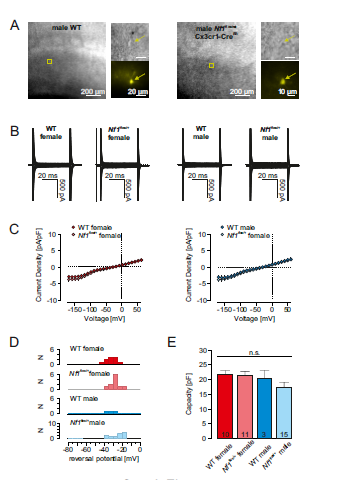

Supplement: Supplementary file 1 — Additional file 1: Supplementary Figures. [file 40478_2023_1525_MOESM1_ESM.docx]
